# Hypoxia-inducible factor-1α promotes macrophage functional activities in protecting hypoxia-tolerant large yellow croaker (*Larimichthys crocea*) against *Aeromonas hydrophila* infection

**DOI:** 10.3389/fimmu.2024.1410082

**Published:** 2024-08-02

**Authors:** Yibo Zhang, Xuelei Wang, Zhenyu Gao, XuJie Li, Ran Meng, Xiongfei Wu, Jie Ding, Weiliang Shen, Junquan Zhu

**Affiliations:** ^1^ Key Laboratory of Aquacultural Biotechnology Ministry of Education, and Key Laboratory of Marine Biotechnology of Zhejiang Province, College of Marine Sciences, Ningbo University, Ningbo, Zhejiang, China; ^2^ Zhejiang Key Laboratory of Aquatic Germplasm Resources, Ningbo Academy of Oceanology and Fishery, Ningbo, Zhejiang, China

**Keywords:** *Larimichthys crocea*, macrophages, metabolic reprogramming, glycolysis, immunity

## Abstract

The immune system requires a high energy expenditure to resist pathogen invasion. Macrophages undergo metabolic reprogramming to meet these energy requirements and immunologic activity and polarize to M1-type macrophages. Understanding the metabolic pathway switching in large yellow croaker (*Larimichthys crocea*) macrophages in response to lipopolysaccharide (LPS) stimulation and whether this switching affects immunity is helpful in explaining the stronger immunity of hypoxia-tolerant *L. crocea*. In this study, transcript levels of glycolytic pathway genes (*Glut1* and *Pdk1*), mRNA levels or enzyme activities of glycolytic enzymes [hexokinase (HK), phosphofructokinase (PFK), pyruvate kinase (PK), and lactate dehydrogenase A (LDHA)], aerobic respiratory enzymes [pyruvate dehydrogenase (PDH), isocitrate dehydrogenase (IDH), and succinate dehydrogenase (SDH)], metabolites [lactic acid (LA) and adenosine triphosphate (ATP)], levels of bactericidal products [reactive oxygen species (ROS) and nitric oxide (NO)], and transcripts and level changes of inflammatory factors [IL1β, TNFα, and interferon (IFN) γ] were detected in LPS-stimulated *L. crocea* head kidney macrophages. We showed that glycolysis was significantly induced, the tricarboxylic acid (TCA) cycle was inhibited, and metabolic reprogramming occurred, showing the Warburg effect when immune cells were activated. To determine the potential regulatory mechanism behind these changes, *Lc*HIF-1α was detected and found to be significantly induced and transferred to the nucleus after LPS stimulation. *LcHif-1α* interference led to a significant reduction in glycolytic pathway gene transcript expression, enzyme activity, metabolites, bactericidal substances, and inflammatory factor levels; a significant increase in the aerobic respiration enzymes; and decreased migration, invasion, and phagocytosis. Further ultrastructural observation by electron microscopy showed that fewer microspheres contained phagocytes and that more cells were damaged after *LcHif-1α* interference. *LcHif-1α* overexpression *L. crocea* head kidney macrophages showed the opposite trend, and promoter activities of *Ldha* and *Il1β* were significantly enhanced after *LcHif-1α* overexpression in HEK293T cells. Our data showed that *Lc*HIF-1α acted as a metabolic switch in *L. crocea* macrophages and was important in polarization. Hypoxia-tolerant *L. crocea* head kidney showed a stronger Warburg effect and inhibited the TCA cycle, higher metabolites, and bactericidal substance levels. These results collectively revealed that *LcHif-1α* may promote the functional activities of head kidney macrophages in protecting hypoxia-tolerant *L. crocea* from *Aeromonas hydrophila* infection.

## 1 Introduction

The large yellow croaker (*Larimichthys crocea*) is one of the most economically valuable marine teleost fishes in aquaculture on the southeast coast of China (1). Natural or artificial breeding conditions, such as summer high temperatures or increased culture density, even lead to large areas with low dissolved oxygen (DO) levels (2, 3). Hypoxia has become the main obstacle to the development of *L. crocea*’s sustainable mariculture (4). In order to effectively solve this problem, the screening of the hypoxia-tolerant *L. crocea* population was carried out (5). Interestingly, the survival rate of the screened hypoxia-tolerant population (T) was higher than normal upon bacterial infection in large-scale cage farming. Similar significant differences in survival rate also appeared after being infected by *Aeromonas hydrophila*, a pathogen that can cause huge economic losses in cultured *L. crocea* (6). Therefore, we generated an interest in why the T has a higher survival rate. Then, it was necessary to find the connection between hypoxia tolerance and resistance to bacteria. As we all know, metabolic transformation occurs after hypoxia. Metabolism has been increasingly considered to play a vital role in regulating immunity (7).

Metabolism is a dynamic process that fuels biological programs by reprogramming them to meet the energy demands. Macrophages are a multiphase population and are important in innate immunity (8). In the early stage of infection, resting macrophages are triggered to polarize into M1-type macrophages [such as lipopolysaccharide (LPS) activation] (9). To meet the high energy and biosynthesis requirements of M1 macrophages, a metabolic transformation occurs in macrophages from oxidative phosphorylation to aerobic glycolysis to rapidly produce adenosine triphosphate (ATP). This also regulates the synthesis of inflammatory factors and provides key metabolites such as reactive oxygen species (ROS), nitric oxide (NO), and lactic acid (LA), for the macrophages to kill pathogens (10–12).

The main driving force of this metabolic adaptation in macrophages was appreciated with the discovery of the immune regulatory roles of the transcriptional regulator hypoxia-inducible factor 1 α (HIF-1α) (13). HIF-1α is easily degraded under normal oxygen tensions; however, it can stably translocate to the nucleus and dimerize with the HIF-1β subunit to drive the transcription of target genes under hypoxia (14). The reported target genes to be upregulated include the glycolytic pathway genes, glucose transporter protein 1 (Glut1), the irreversible enzymes (Hk, Pfk, and Pkm), and lactate dehydrogenase (Ldha), which promotes the conversion of pyruvate to lactate, as well as Pdk1, which represses the expression of pyruvate dehydrogenase (PDH). Aerobic respiratory enzymes can be downregulated, such as PDH, which is responsible for the conversion of pyruvate to acetyl coenzyme A (acetyl-CoA) into the tricarboxylic acid cycle (TCA cycle), as well as the enzymes isocitrate dehydrogenase (IDH) and succinate dehydrogenase (SDH) in the TCA cycle (15).

Recent studies have elucidated that HIF-1α plays a vital role in innate immune response (16, 17). Pathogens or LPS can induce the accumulation of HIF-1α in macrophages through transcription and translation activation, which is independent of hypoxia (18). HIF-1α can also activate interleukin (IL) 1β gene expression and some inflammatory factors and affect the production of bactericidal substances such as LA, NO, and ROS (7, 19, 20). In the absence of HIF-1α, the consequent decrease in the rate of glycolysis and energy production causes the migration, invasion, and phagocytosis of macrophages to be impaired (21). Mice with immune cell-specific deletion of HIF-1α showed impaired inflammatory responses with a lower survival rate (16).

Although the necessity of HIF-1α for immune cell metabolism and polarization has been studied in mammals, it is only in recent years that it was found that fish macrophages can be polarized, and the role of HIF-1α in the reprogramming and polarization of macrophage metabolism in marine teleost species has been rarely reported (22). In our previous research, some genes with significant single-nucleotide polymorphism of hypoxia tolerance *L. crocea* were related to HIF-1α translation or protein stabilization (5). Further, transcriptome sequencing of the head kidney of *L. crocea* infected with *A. hydrophila* showed that the mRNA expression level of *Hif-1α* in T was significantly higher (23). In view of the important role of HIF-1α in metabolism and immunity, we are interested in the role of HIF-1α in the infection of hypoxia-tolerant *L. crocea* by *A. hydrophila* (24–26). Therefore, in this study, we investigated whether metabolic reprogramming occurs in *L. crocea* head kidney macrophages under LPS stimulation, the role of HIF-1α in metabolic reprogramming and polarization, and the response of HIF-1α to *A. hydrophila* in the hypoxia-tolerant *L. crocea* in order to preliminarily elucidate whether HIF-1α promotes macrophage activities in the protection of large yellow croaker against bacterial infection.

Our study will help clarify the immune mechanism of aquatic animals against disease outbreaks and improve the understanding of metabolic checkpoints that control immunity. This may provide new insights into the metabolism and immune defense mechanisms of marine teleosts and provide a theoretical basis and scientific evidence for the germplasm superiority of the new strain of hypoxia-tolerant large yellow croaker.

## 2 Materials and methods

### 2.1 Ethics statement

The large yellow croaker (*L. crocea*) samples here were commercially cultured animals, and all the experiments followed the requirements of animal ethics and were approved by the Experimental Animal Research Center of Ningbo University.

### 2.2 Fish experiments and sample collection

In previous experiments, the 24-h median lethal dose (LD50) for *A. hydrophila* infection in *L. crocea* was determined to be 8 × 10^6^ CFU/mL (0.5 mL) (DO 7.5–8.0 mg/L, 23°C ± 0.5°C) (23), and therefore, in the present experiments, this dose injection was used for both groups. Healthy 40 fish (15.80 ± 1.63 cm in length and 64.61 ± 5.63 g in weight at a random mix of male and female) samples were randomly selected from the two groups for intraperitoneal injections, and the experiment was repeated three times, with the temperature kept the same as the pre-experiment. At 0 h, 3 h, 12 h, and 24 h, one fish from each group was randomly selected in parallel, 0.05% MS-222 (3-aminobenzoic acid ethyl ester methanesulfonate; Sigma-Aldrich Corp., St. Louis, MO, USA) was used as euthanasia agent, and the head kidney was collected. The acquired head kidney was immediately transferred into RNase-free tubes, frozen in liquid nitrogen, and stored at −80°C for Western blotting and determination of biochemical indices. Protein concentrations of the head kidney were measured using the BCA Protein Assay Kit (Sangon, Shanghai, China) in accordance with the manufacturer’s instructions.

### 2.3 Cell culture and LPS challenge

*L. crocea* head kidney macrophages were obtained from the *L. crocea* head kidney macrophage cell line established by Cui et al. (8). *L. crocea* head kidney macrophages were cultured in Dulbecco’s Modified Eagle Medium/Nutrient Mixture-F12 (DMEM/F12, Biological Industries, Beit HaEmek, Israel) containing 15% fetal bovine serum (FBS; Gibco, Grand Island, NY, USA), and 100 IU/mL penicillin-streptomycin (Gibco, USA) at 28°C.

For the LPS challenge, LPS (#L2880, Sigma-Aldrich) was diluted to a final concentration of 5 mg/mL in phosphate-buffered saline (PBS). Cells (1 × 10^6^) were plated on six-well plates to form a complete monolayer, and LPS (50 μg/mL) was then added. The control group was treated with the same amount of PBS only.

### 2.4 RNA extraction, reverse transcription, and real-time fluorescence-based quantitative PCR analysis

RNA from *L. crocea* head kidney macrophages was extracted using an Omega RNA extraction kit (Omega, Doraville, GA, USA). RNA integrity and purity were assessed using 1% agarose gel electrophoresis and NanoDrop spectrophotometry (Allsheng, Hangzhou, China), respectively. The RNA was reverse-transcribed into cDNA using the PrimeScript™ RT reagent Kit (TaKaRa, Dalian, China) as a template for real-time fluorescence-based quantitative PCR analysis, followed by quantitative PCR using a Roche Light Cycler 480 II with SYBR Green Master I (Roche, Basel, Switzerland). The primers used for real-time quantitative PCR (RT-qPCR) are listed in **Supplementary Table S1**.

### 2.5 Small interfering RNA-mediated RNA silencing *in vitro*

Specific mall interfering RNAs (siRNAs) targeting *LcHif-1α* (*siLcHif-1α*) were synthesized by GenePharma (Shanghai, China) (**Supplementary Table S1**). Small interfering negative control (siNC) was used as a negative control. si*LcHif-1α* and siNC were mixed with Lipo6000 (Beyotime, Shanghai, China), added into one well of a six-well plate with 6 × 10^5^ *L. crocea* head kidney macrophages, cultured for 12 h, and then incubated with LPS for 12 h. The transfection efficiency was analyzed by flow cytometry (**Supplementary Figure S1**). After LPS incubation for 12 h, RT-qPCR and Western blotting assays were used to detect the interference efficiency.

### 2.6 Overexpression vector construction and adenovirus infection

The open reading frames of *LcHif-1α* were amplified using specific primers (**Supplementary Table S1**) and inserted into the vector pcDNA3.1 to construct the oe*LcHif-1α* overexpression vectors. The construction and packaging of the adenovirus vector were completed by HANBIO Co., Ltd (Hubei, China). Adenovirus exists widely in the animal kingdom including fish (27), and studies have shown that it is feasible to overexpress genes in fish with adenovirus (28). In this study, Adeasy adenovirus (Ad5) system was used to package the virus. After the shuttle plasmid (pAdEasy-EF1-MCS-CMV) was inserted into the open reading frame of LcHif-1α, it was linearized. The recovered linearized shuttle plasmid was transferred into competent cells of *Escherichia coli* BJ5183 containing adenovirus skeleton plasmid pAdeasy-1 for intracellular recombination. After verification, the correct recombinant plasmid was transformed into competent *E. coli* stbl3, and the recombinant plasmid with high purity was obtained. After the plasmid vector was extracted with high purity and without endotoxin, the plasmid was transfected into 293A cells with Lipofiter™ transfection reagent, and then the virus was collected to obtain Adeasy-LcHif-1α and Adeasy-NC. Adenoviral infection was performed according to the recommended protocol. In total, 2 × 10^10^ PFU/mL of overexpression adenoviruses and 4.5 × 10^10^ PFU/mL of control adenoviruses were quantified and diluted in serum-free DMEM/F12. Subsequently, the adenovirus mixtures were added to the cultured plates containing *L. crocea* head kidney macrophages with a 50% volume of fresh culture solution. Adenovirus infection was continued for 4 h and then replenished to the normal culture volume. After 12 h, incubation was continued using a fresh complete medium. After another 12 h, LPS was added and incubated. The transfection efficiency was analyzed by flow cytometry (**Supplementary Figure S1**). RT-qPCR and Western blotting assays were used to detect *LcHif-1α* expression after 12 h incubation with the LPS.

### 2.7 Western blotting

Specific primers were used to amplify *Lc*HIF-1α in the open reading frame (265–972 bp) (**Supplementary Table S1**). The amplified PCR product was linked to the PEASY-blunt E1 expression vector (TransGen Biotechnology, Beijing, China). After sequencing confirmed that the inserted sequence was correct, the competent cell Rosetta (DE3) (TransGen Biotechnology, China) was transformed. DE3 was induced to express a recombinant protein with 0.1 M IPTG as inducer. According to the manufacturer’s instructions, His-tag protein purification kit (Beyotime, Shanghai, China) was used to extract and purify the recombinant protein. The purified recombinant protein was put into a dialysis bag and renaturated in dialysate with a gradient concentration of urea. Rat immunization was carried out as described above (29). The antiserum was extracted for subsequent experiments (**Supplementary Figure S2**).

Radioimmunoprecipitation assay (RIPA) buffer (Solarbio, Beijing, China) with phenylmethane sulfonyl fluoride (PMSF; Solarbio, China) was used to extract total protein from the head kidney or *L. crocea* head kidney macrophages. Protein concentrations of the head kidney or cells were measured using the BCA Protein Assay Kit (Sangon, China) in accordance with the manufacturer’s instructions. Protein samples (20 μg) were separated using 10% sodium dodecyl sulfate–polyacrylamide gel electrophoresis and then transferred to polyvinylidene difluoride (PVDF; Solarbio, China) membranes. Proteins were blocked using 5% skimmed milk powder, followed by overnight incubation with the targeting antibodies at 4°C. After washing thrice with Tris-buffered saline with Tween (TBST; 20 mmol/L Tris-HCl, 150 mmol/L NaCl, and 0.05% Tween-20), the PVDF membranes were incubated with horseradish peroxidase (HRP)-labeled goat anti-rat IgG (A0192, Beyotime Biotechnology, China) or HRP-labeled goat anti-rabbit IgG (D110058, Sangon Biotech, China) for 1 h at 37°C. The immune complexes were visualized using a chemiluminescence imaging analysis system (Tanon 5 200, Tanon, Shanghai, China). Data were normalized to the level of the actin protein. Anti-ACTB antibody (AF5003, Beyotime, China) was used as the reference. Antibodies against COX2 (Cat. No. AF1924) were from Beyotime (China). The protein band densities were quantified using ImageJ software (National Institutes of Health, USA).

### 2.8 Measurement of biochemical indices and enzyme activities of the head kidney and macrophages

The LA, NO, and ATP levels in the head kidney and *L. crocea* head kidney macrophages were measured using biochemical indicator kits (Solarbio, China). The ROS level in macrophages was measured using a Reactive Oxygen Species Assay Kit (Solarbio, China). Hexokinase (HK), pyruvate kinase (PK), phosphofructokinase (PFK), lactate dehydrogenase (LDH), PDH, IDH, and SDH activities were measured using assay kits (Solarbio, China) following the manufacturer’s instructions. The concentration of head kidney IL1β, TNFα, and interferon (IFN) γ were measured using an enzyme-linked immunosorbent assay test kit following the manufacturer’s instructions (Shanghai Duqiao, China). The absorbance of the sample was measured using a SpectraMax M3 multifunctional detection station (Molecular Devices, San Jose, CA, USA).

### 2.9 Immunofluorescence assay of LcHIF-1α

*L. crocea* head kidney macrophages were washed with PBS buffer for 3 min, fixed with 4% paraformaldehyde for 30 min, permeated with 0.3% Triton X-100-PBS for 5 min, incubated with 5% bovine serum albumin in PBS buffer for 1.5 h, and then labeled with 1:500 diluted *Lc*HIF-1α primary antibody. Antibody binding was detected using the secondary antibody conjugated with Cy3 Labeled Goat Anti-Rat IgG(H+L) (1:500, A0507, Beyotime, China). Diamidino-phenyl-indole buffer (DAPI; Beyotime) was used to counterstain the cell nucleus. The samples were examined under a confocal microscope (LSM510, Zeiss, Oberkochen, Germany). Untreated cells, siNC, or control adenoviruses served as the respective controls.

### 2.10 Functional evaluation

#### 2.10.1 Measurement of intracellular ROS

Intracellular ROS fluorescence in macrophages was assayed using a Reactive Oxygen Species Assay kit (Solarbio, China). DCFH-DA was diluted to the working concentration (10 μM) by serum-free DMEM/F12 medium. After transfection with LcHif-1α siRNA, or infection with LcHif-1α adenovirus, and incubation with LPS for 12 h, *L. crocea* head kidney macrophages were washed with PBS, fixed with 4% paraformaldehyde (Solarbio, China) for 30 min, and washed with PBS, and diluted fluorescent probes were added to 1 mL/well of confocal dish. They were incubated in the dark at 28°C for 20 min, and then the nucleus was stained with DAPI and incubated in the dark at 28°C for 5 min. ROS fluorescence was detected under a confocal microscope (Zeiss LSM510, Germany).

#### 2.10.2 Wound healing assay

*L. crocea* head kidney macrophages were seeded in a six-well plate after being transfected with *LcHif-1α* siRNA or infected with the *LcHif-1α* adenovirus. When the cells covered the bottom of the plate, a straight scratch was slowly made in the monolayer across the center of each well with a 10-μL pipette tip. The plate was then gently washed to remove detached cells. The macrophages were cultured in DMEM/F12 (Biological Industries, Israel) containing 2% FBS (Gibco, USA) and 100 IU/mL penicillin-streptomycin (Gibco, USA) for an additional 12 h under LPS challenge. Then, they were observed and photographed using an inverted microscope (Ti-S, Nikon, Tokyo, Japan); the scratched area was visualized and quantified using ImageJ software (National Institutes of Health, USA). Each experiment was repeated three times. siNC or control adenovirus-transfected cells were used as the respective negative controls.

#### 2.10.3 Chemotaxis assay *in vitro*

The *in vitro* chemotaxis test was carried out using a 24-well Costar Transwell apparatus (Corning Company, USA). A complete medium (500 μL with 20% FBS) was added to the lower chamber of the Transwell unit. Polycarbonate filters with a pore diameter of 8 μm were then placed into the lower wells, and 100 μL *L. crocea* head kidney macrophage suspension containing 5 × 10^4^ cells transfected with *LcHif-1α* siRNA, infected with the *LcHif-1α* adenovirus, or the negative control was added to the upper chamber and incubated with LPS at 28°C for 12 h. Then, the polycarbonate membrane was removed, fixed in 4% paraformaldehyde, and stained with 0.1% crystal violet aqueous solution (Solarbio, China). The cells on the upper surface of the membrane were wiped off and transferred to a slide glass. Xylene was added dropwise, and the slides were sealed with neutral resin and then observed under a microscope (×400) (Nikon, Japan). The number of migratory cells was counted using ImageJ software (National Institutes of Health, USA). Each experiment was repeated three times.

#### 2.10.4 Phagocytosis

*L. crocea* head kidney macrophages with *LcHif-1α* siRNA, infected with the *LcHif-1α* adenovirus, or the negative control were incubated with fluorescent beads (Tianjin Goose Technology China) and LPS for 12 h. The ratio of fluorescent beads to cells was 50:1. The phagocytic ability was observed using flow cytometry (Becton Dickinson, Franklin Lakes, NJ, USA) under 488-nm excitation. The acquired data were analyzed using FlowJo v10 software (Ashland, OR, USA).

#### 2.10.5 Electron microscopy

*L. crocea* head kidney macrophages with si*LcHif-1α* or siNC were incubated with LPS and beads (Tianjin Goose Technology, China) for 12 h, the culture medium was discarded, and 2.5% glutaraldehyde was added and fixed for 5 min. The cells were scraped off slowly and lightly along one direction with a cell scraper, collected in a centrifuge tube with a pap pipette, and centrifuged at 800 ×*g* for 2 min, and the supernatant was discarded and added with 2.5% glutaraldehyde. The cell mass was suspended in 2.5% glutaraldehyde, fixed overnight at 4°C in the dark, then fixed in 1% osmic acid for 1–2 h, and dehydrated in an acetone gradient. Finally, the cells were stained with uranyl acetate and lead citrate and observed via electron microscopy.

### 2.11 Plasmid construction and dual-luciferase reporter assay

Using genomic DNA as a template, the *LcLdha* and *LcIl1β* gene promoter fragments (**Supplementary Table S1**) were amplified with specific primers and inserted into a pGL3-basic vector to construct luciferase reporter vectors, respectively: pGL3-proma-*LcLhda* and pGL3-proma-*LcIl1β*. For the luciferase assays, reporter plasmids, expression plasmids, and pRT-TK Renilla luciferase plasmids were co-transfected into HEK293T cells using Lipo6000 (Beyotime, China) (12, 30). After 24 h of transfection, cells were harvested and detected using the Dual-Luciferase Reporter Assay System (Vazyme, Nanjing, China). The firefly and Renilla luciferase activities were read using a SpectraMax M3 multifunctional detection station (Molecular Devices, USA). Transfections were performed in triplicate, with three parallels per transfection (n = 9).

### 2.12 Statistical analysis

Statistical analyses were performed using SPSS 19.0 (IBM, Armonk, NY, USA). All data are shown as the mean ± standard error of the mean. Analysis of variance was used to determine the significance of differences between groups. Statistical significance was defined as *p* < 0.05.

## 3 Results

### 3.1 mRNA expression of metabolic and immune-related genes changed in *L. crocea* head kidney macrophages after LPS induction

**Figure 1:**
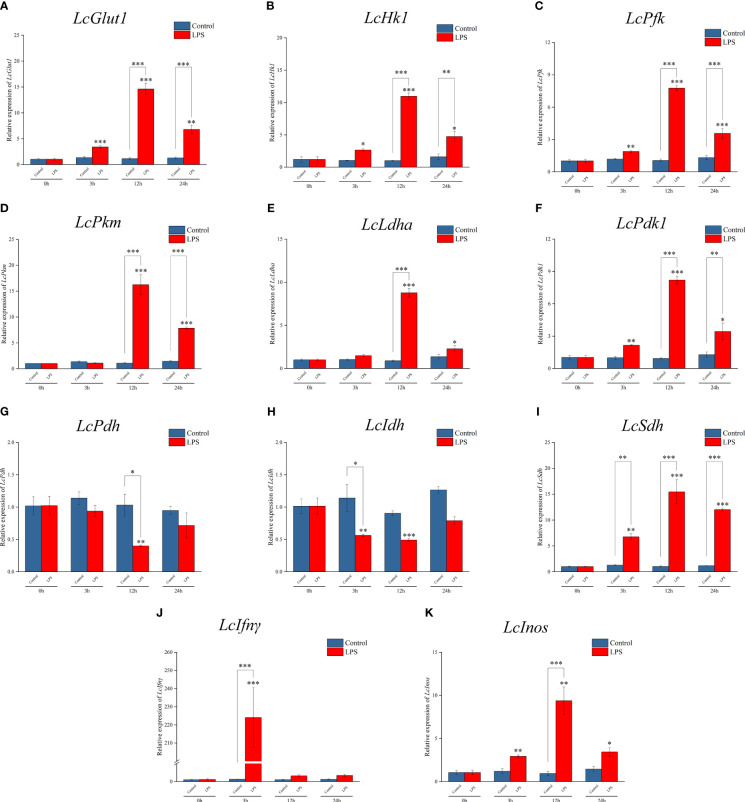
Expression patterns of metabolic and immune-related genes in *Larimichthys crocea* head kidney macrophages after LPS stimulation. mRNA expressions of *LcGlut1* **(A)**, *LcHk1* **(B)**, *LcPfk* **(C)**, *LcPkm* **(D)**, *LcLdha* **(E)**, *LcPdk1* **(F)**, *LcPdh* **(G)**, *LcIdh* **(H)**, *LcSdh* **(I)**, *LcIfnγ* **(J)**, and *LcInos* **(K)** in macrophages at 0 h, 3 h, 12 h, and 24 h following LPS stimulation or the control (n = 3). LPS, lipopolysaccharide. “*” indicates P < 0.05, “**” indicates P < 0.01, and “***” indicates P < 0.001.

RT-qPCR detection found that the mRNA expressions of *LcGlut1*, *LcHk1*, *LcPfk*, *LcPkm*, *LcLdha*, and *LcPdk1* increased significantly at 3 h or 12 h relative to the control group following exposure to LPS (**Figures 1A–F**). There was a decrease in the expressions of *LcPdh* and *LcIdh* at 3 h or 12 h (**Figures 1G, H**), whereas there was an increase in *LcSdh* (**Figure 1I**). LPS promoted a significant increase in *LcIfnγ* and *LcInos* at 3 h or 12 h (**Figures 1J, K**). These findings indicate that the metabolic pathway changes to glycolysis and cell polarization to M1 type after LPS induction.

### 3.2 *Lc*HIF-1α and *Lc*COX2 proteins were induced in *L. crocea* head kidney macrophages by LPS

**Figure 2:**
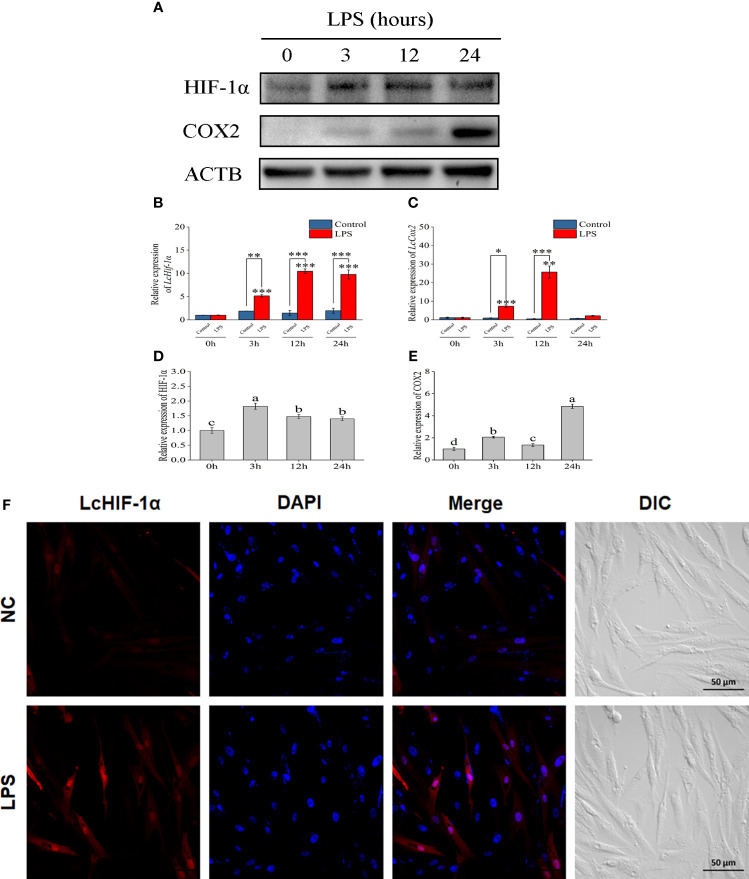
Changes in *Lc*HIF-1α and *Lc*COX2 protein in *Larimichthys crocea* head kidney macrophages after LPS stimulation. Changes in the level of *Lc*HIF-1α **(A, B, D)** and *Lc*COX2 **(A, C, E)** mRNA (n = 3) and protein expression in macrophages after 0-, 3-, 12-, and 24-h incubation with LPS. Subcellular localization of *Lc*HIF-1α in macrophages exposed to LPS for 12 h **(F)**. LPS, lipopolysaccharide. “*” indicates P < 0.05,“**” indicates P < 0.01, and “***” indicates P < 0.001. The different letters indicate significant differences (P < 0.05).

Through the Western blotting experiment, *Lc*HIF-1α and *Lc*COX2 were observed significantly increased by LPS stimulation at 3 h and up to 24 h (**Figures 2A–E**). Confocal observation showed that *L. crocea* head kidney macrophages were spindle-shaped, which was consistent with the microscopic observation results in previous studies (8). *Lc*HIF-1α protein increased significantly and entered the nucleus at a higher rate after 12 h of LPS stimulation (**Figure 2F**). These results indicate that *Lc*HIF-1α may enter the nucleus, regulating the transcription of downstream genes.

### 3.3 The activity of key metabolic enzymes, levels of metabolites, and inflammatory factors changed in *L. crocea* head kidney macrophages after LPS stimulation

**Figure 3:**
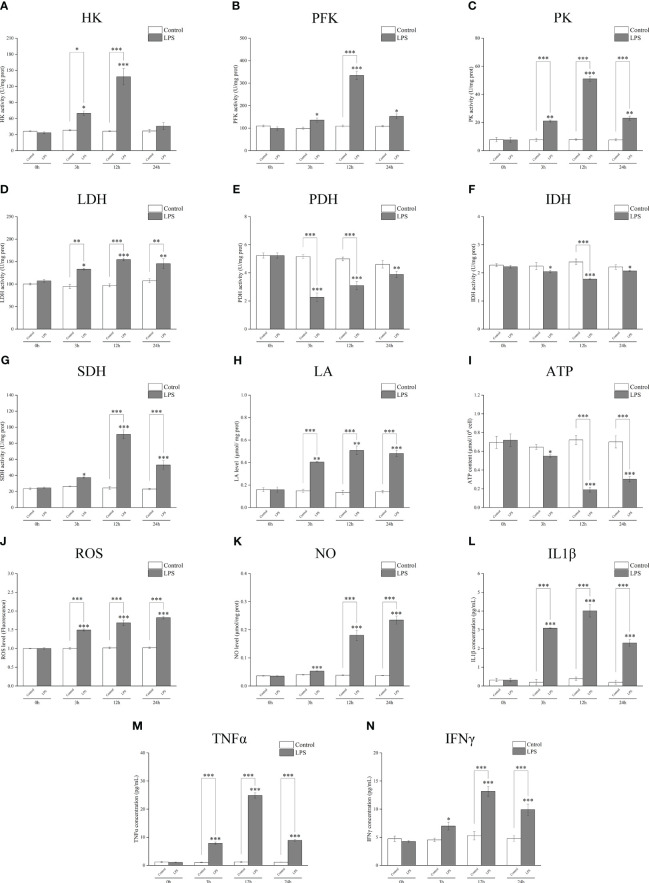
Detection of the activities of the key metabolic enzymes and levels of metabolites and inflammatory factors in *Larimichthys crocea* head kidney macrophages after LPS stimulation. Changes in activities of HK **(A)**, PFK **(B)**, PK **(C)**, LDH **(D)**, PDH **(E)**, IDH **(F)**, and SDH **(G)** and levels of LA **(H)**, ATP **(I)**, ROS **(J)**, NO **(K)**, IL1β **(L)**, TNFα **(M)**, and IFNγ **(N)** in macrophages after 0 h, 3 h, 12 h, and 24 h of LPS incubation (n = 3). LPS, lipopolysaccharide; HK, hexokinase; PFK, phosphofructokinase; PK, pyruvate kinase; LDH, lactate dehydrogenase; PDH, pyruvate dehydrogenase; IDH, isocitrate dehydrogenase; SDH, succinate dehydrogenase; LA, lactic acid; ATP, adenosine triphosphate; ROS, reactive oxygen species; NO, nitric oxide. “*” indicates P < 0.05,“**” indicates P < 0.01, and “***” indicates P < 0.001.

A biochemical kit test showed that the activities of the key metabolic enzymes HK, PFK, PK, and LDH significantly increased after incubation with LPS for 3 h or 12 h (**Figures 3A–D**); PDH and IDH activities significantly decreased (**Figures 3E, F**); SDH activity increased (**Figure 3G**). LPS stimulation induced an increase in the glycolysis metabolite LA (**Figure 3H**), whereas the ATP yield was reduced (**Figure 3I**). The levels of ROS and NO (**Figures 3J, K**) and those of inflammatory factors IL1β, TNFα, and IFNγ significantly increased after incubation with LPS (**Figures 3L–N**). These findings further suggest that the metabolic pathway changes to glycolysis and cell polarization to M1 type after LPS induction.

### 3.4 *Lc*Hif-1α interference or overexpression influenced the levels of *LcHif-1α* and *LcCox2* in *L. crocea* head kidney macrophages

**Figure 4:**
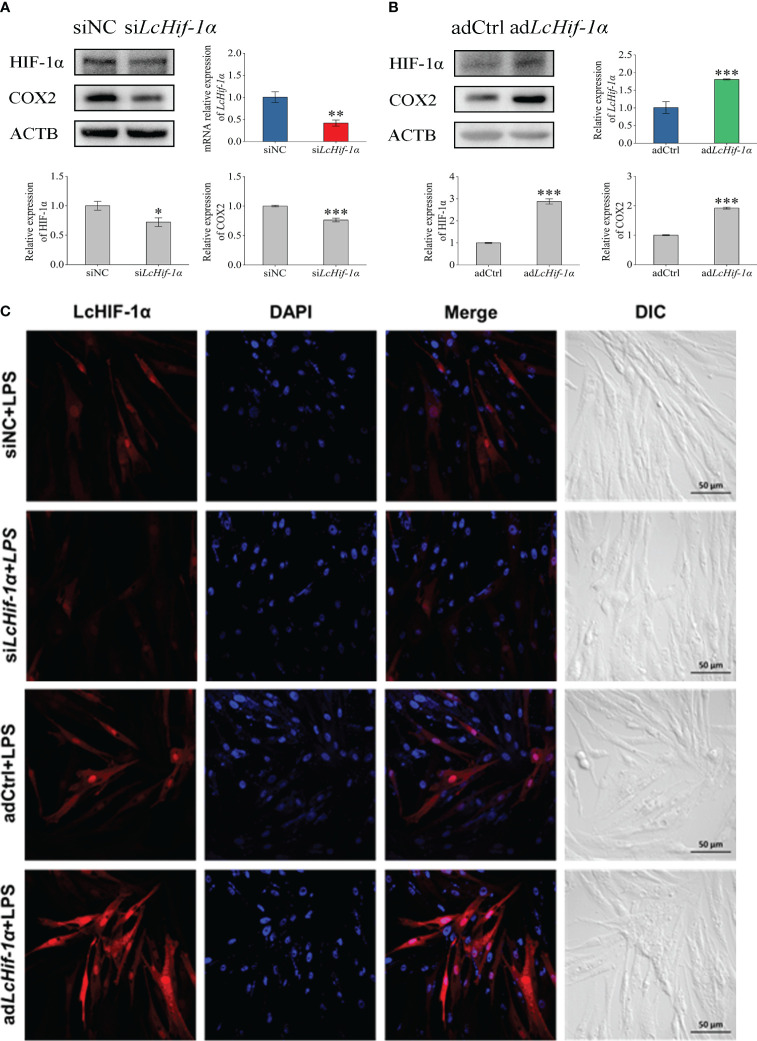
Changes in expressions of *Lc*HIF-1α and *Lc*COX2 in *Larimichthys crocea* head kidney macrophages after *LcHif-1α* interference or overexpression following 12-h LPS stimulation. Changes in level of *LcHif-1α* mRNA expression (n = 3) and *Lc*HIF-1α and *Lc*COX2 protein expression in macrophages after *LcHif-1α* interference **(A)** or overexpression **(B)** following 12-h LPS stimulation. Subcellular localization of *Lc*Hif-1α in macrophages after *LcHif-1α* interference or overexpression following 12-h LPS stimulation **(C)**. DIC, differential interference contrast; LPS, lipopolysaccharide. “*” indicates P < 0.05,“**” indicates P < 0.01, and “***” indicates P < 0.001.

The results of the Western blotting experiment showed that *Lc*HIF-1α and *Lc*COX2 significantly decreased after *LcHif-1α* interference or significantly increased after *LcHif-1α* overexpression following LPS stimulation for 12 h (**Figures 4A, B**). Confocal observation showed that *Lc*HIF-1α protein decreased significantly and entered the nucleus at a lower rate after interference and showed increased *Lc*HIF-1α protein and a higher entered nucleus rate after overexpression (**Figure 4C**). These results show that after *Lc*Hif-1α is successfully interfered with or overexpressed, its nuclear regulation of downstream genes is affected, and the protein expression of COX2 is regulated by *Lc*HIF-1α.

### 3.5 *L. crocea* head kidney macrophage *LcHif-1α* influenced the mRNA expression of metabolic and immune-related genes

**Figure 5:**
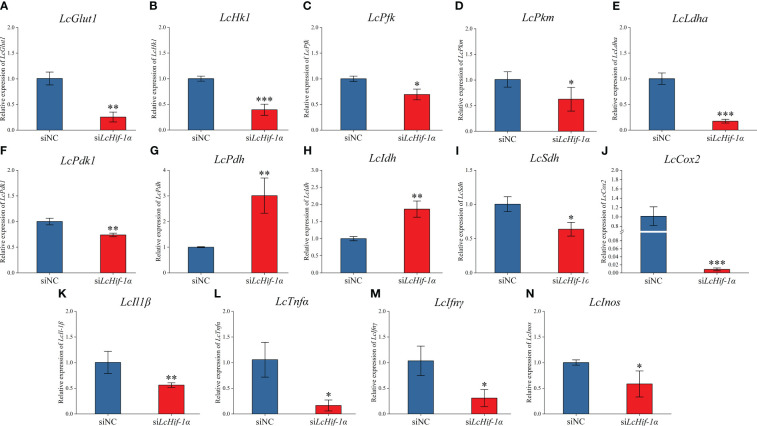
Expression patterns of metabolic and immune-related genes in *Larimichthys crocea* head kidney macrophages after *LcHif-1α* interference following 12-h LPS stimulation. mRNA expressions of *LcGlut1* **(A)**, *LcHk1* **(B)**, *LcPfk* **(C)**, *LcPkm* **(D)**, *LcLdha* **(E)**, *LcPdk1* **(F)**, *LcPdh* **(G)**, *LcIdh* **(H)**, *LcSdh* **(I)**, *LcCox2* **(J)**, *LcIl1β* **(K)**, *LcTnfα* **(L)**, *LcIfnγ* **(M)**, and *LcInos* **(N)** in macrophages after *LcHif-1α* interference following 12-h LPS stimulation (n = 3). LPS, lipopolysaccharide. “*” indicates P < 0.05,“**” indicates P < 0.01, and “***” indicates P < 0.001.

RT-qPCR detection showed that mRNA expressions of *LcGlut1*, *LcHk1*, *LcPfk*, *LcPkm*, *LcLdha*, and *LcPdk1* (**Figures 5A–F**) significantly reduced after *LcHif-1α* interference following LPS stimulation for 12 h. *LcHif-1α* interference induced *LcPdh* and *LcIdh* (**Figures 5G, H**) mRNA expression, whereas it inhibited *LcSdh* (**Figure 5I**). The pro-inflammatory genes *LcIl1β*, *LcTnfα*, *LcIfnγ*, and *LcInos* (**Figures 5K–N**) were lower than in the siNC, as were the expressions of the M1-type macrophage marker gene *LcCox2* after *LcHif-1α* interference following 12-h LPS stimulation (**Figure 5J**). *Lc*Hif-1α overexpressed macrophages showed the opposite trend (**Supplementary Figure S3**). These findings indicate that the metabolic pathway change is regulated by *Lc*Hif-1α, which promotes the transformation from metabolism to glycolysis and promotes the polarization of macrophages to the M1 type.

### 3.6 *LcHif-1α* influenced the activity of the key metabolic enzymes and levels of metabolites and inflammatory factors in *L. crocea* head kidney macrophages

**Figure 6:**
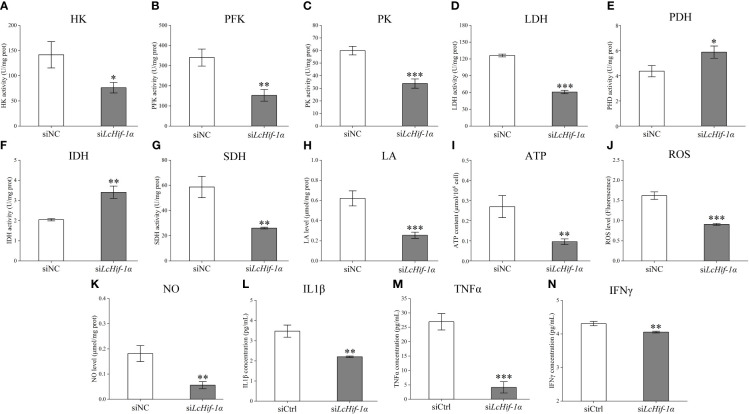
Detection of the activities of the key metabolic enzymes and levels of metabolites and inflammatory factors in *Larimichthys crocea* head kidney macrophages after *LcHif-1α* interference following 12-h LPS stimulation. Changes in activities of HK **(A)**, PFK **(B)**, PK **(C)**, LDH **(D)**, PDH **(E)**, IDH **(F)**, and SDH **(G)** and levels of LA **(H)**, ATP **(I)**, ROS **(J)**, NO **(K)**, IL1β **(L)**, TNFα **(M)**, and IFNγ **(N)** in macrophages after *LcHif-1α* interference following 12-h LPS stimulation (n = 3). LPS, lipopolysaccharide; HK, hexokinase; PFK, phosphofructokinase; PK, pyruvate kinase; LDH, lactate dehydrogenase; PDH, pyruvate dehydrogenase; IDH, isocitrate dehydrogenase; SDH, succinate dehydrogenase; LA, lactic acid; ATP, adenosine triphosphate; ROS, reactive oxygen species; NO, nitric oxide. “*” indicates P < 0.05,“**” indicates P < 0.01, and “***” indicates P < 0.001.

Biochemical kit test results showed that the activities of the glycolytic pathway enzymes HK, PFK, PK, and LDH significantly reduced after *LcHif-1α* interference (**Figures 6A–D**), and the activities of the aerobic respiratory enzymes PDH and IDH significantly increased (**Figures 6E, F**), whereas SDH significantly decreased (**Figure 6G**). The glycolytic pathway metabolic product LA and ATP levels decreased after *LcHif-1α* interference (**Figures 6H, I**). The levels of the bactericidal substances ROS, NO, and inflammatory factors IL1β, TNFα, and IFNγ also decreased significantly after *LcHif-1α* interference (**Figures 6J–N**). There showed an opposite trend in *Lc*Hif-1α overexpressed macrophages (**Supplementary Figure S4**). These findings further suggest the metabolic pathway change is regulated by *Lc*Hif-1α, which promotes the polarization of macrophages to the M1 type.

### 3.7 *LcHif-1α* influenced the *L. crocea* head kidney macrophage functional activity

**Figure 7:**
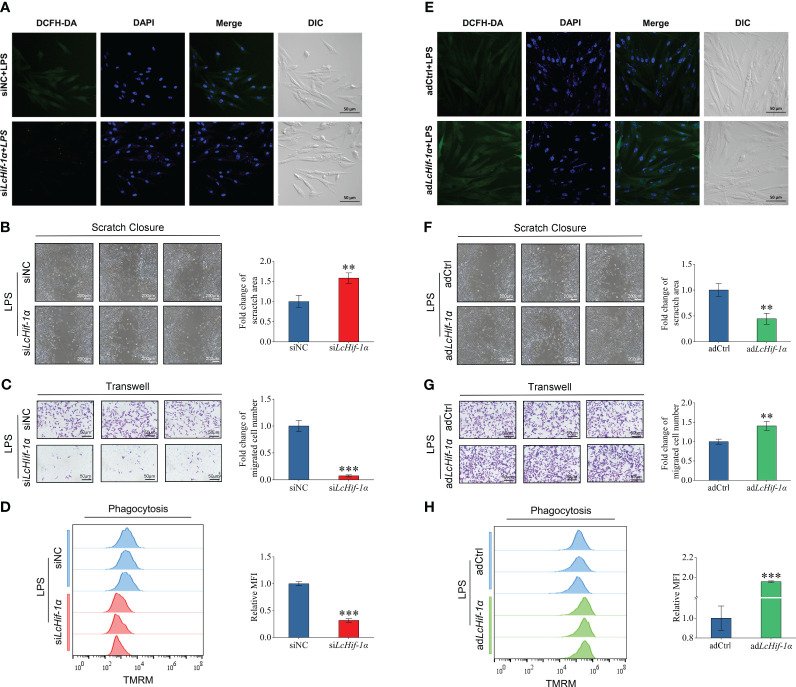
Functional activity of *Larimichthys crocea* head kidney macrophages after *LcHif-1α* interference following 12-h LPS stimulation. Laser confocal microscopy analysis of ROS change in macrophages after *LcHif-1α* interference following 12-h LPS stimulation **(A)**. Analysis of migration **(B)**, invasion **(C)**, and phagocytosis **(D)** of macrophages after *LcHif-1α* interference or overexpression **(E–H)** following 12-h LPS stimulation (n = 3). DCFH-DA, dichloro-dihydro-fluorescein diacetate; DIC, differential interference contrast; MFI, mean fluorescence intensity; TMRM, tetramethyl rhodamine methyl ester; LPS, lipopolysaccharide; ROS, reactive oxygen species. “*” indicates P < 0.05,“**” indicates P < 0.01, and “***” indicates P < 0.001.

**Figure 8:**
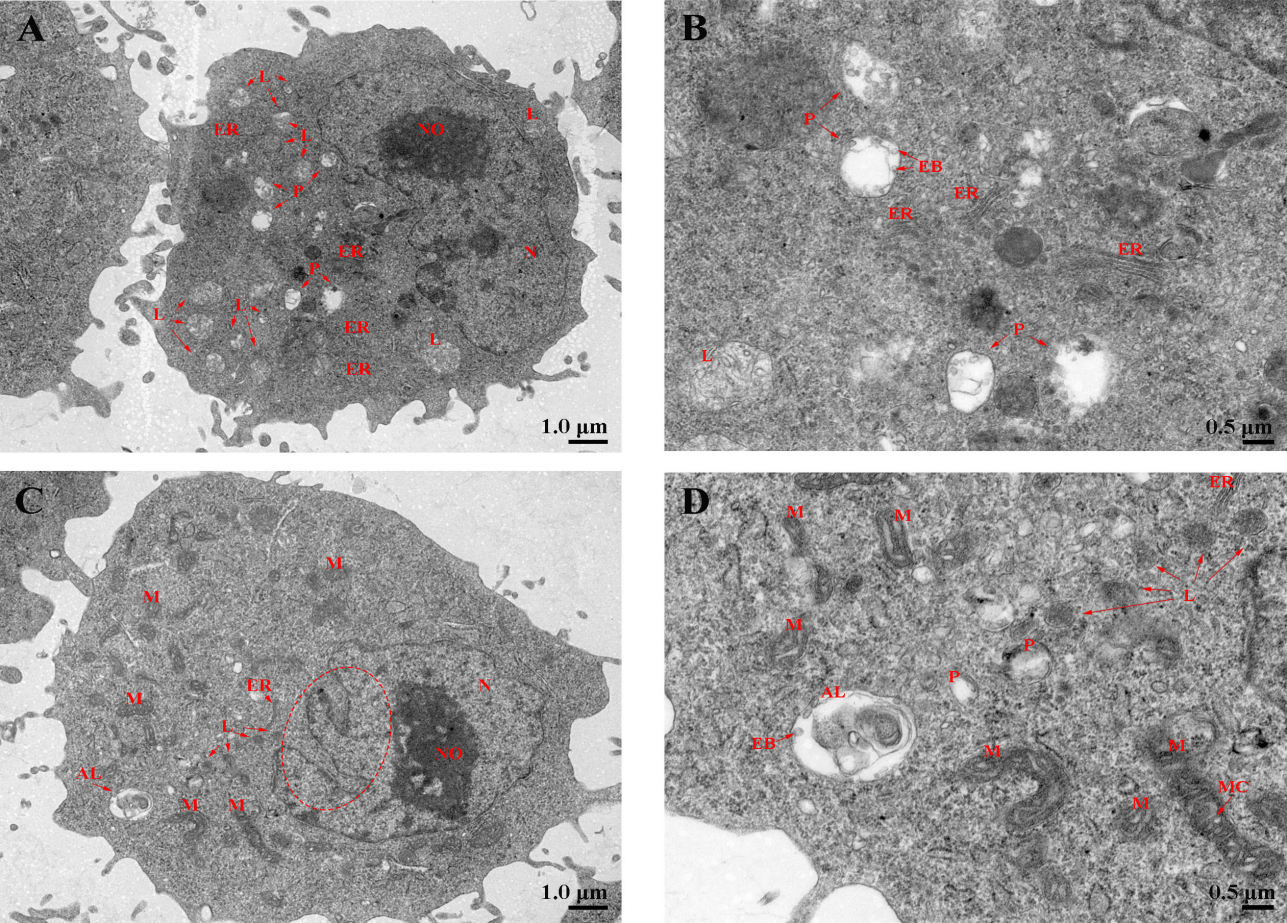
Phagocytosis of *Larimichthys crocea* head kidney macrophages after *LcHif-1α* interference following 12-h LPS stimulation. **(A, B)** The ultrastructure of *L. crocea* head kidney macrophages transfected with siNC and stimulated by LPS for 12 h. **(C, D)** The ultrastructure of *L. crocea* head kidney macrophages transfected with si*LcHif-1α* and stimulated by LPS for 12 h. N, nucleus; NO, nucleoli; L, lysosome; P, phagocyte; AL, autolysosome; M, mitochondria; MC, mitochondrial crest; ER, endoplasmic reticulum; EB, phagocytic microsphere. The red dashed ellipse shows the concave nuclear membrane. LPS, lipopolysaccharide.

Laser confocal microscopy analysis shows that the macrophage bactericidal substance ROS reduced after *LcHif-1α* interference (**Figure 7A**). The wounding healing assay, Transwell, and flow cytometry analysis showed that the migration, invasion, and phagocytosis of cells were significantly reduced after *LcHif-1α* interference (**Figures 7B–D**). Macrophage functional activity was significantly induced after *LcHif-1α* overexpression (**Figures 7E–H**). The ultrastructure of *L. crocea* head kidney macrophage after *Lc*Hif-1α interference is shown in **Figure 8**. There were many folds and protrusions on the cell surface showing the unique macrophage morphology, and the nucleus was deeply colored (**Figures 8A–D**). After the interference of *LcHif-1α*, the nuclear membrane was seriously depressed inward; mitochondria were elongated and folded; some mitochondrial ridges were reduced or disappeared; and fewer phagocytes, lysosomes, and endoplasmic reticulum, and autolysosome appeared, with a few phagocytic microspheres inside (**Figures 8C, D**). In the siNC control group, the morphology of the nuclear membrane was regular, and the cytoplasm contained a large number of lysosomes and phagocytes, with abundant endoplasmic reticulum and more microspheres in phagocytes (**Figures 8A, B**). The above results show that *LcHif-1α* influenced *L. crocea* head kidney macrophage functional activity.

### 3.8 *LcHif-1α* overexpression increased the promoter activities of *Lc*Ldha and *Lc*Il1β

**Figure 9:**
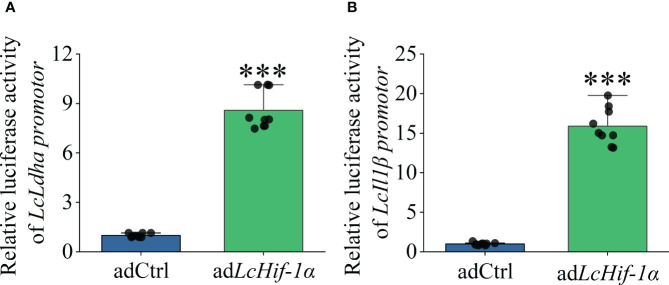
Changes in transcriptional activities of *LcLdha* and *LcIl1β* promoters after *LcHif-1α* overexpression of HEK293T. Changes in transcription activity of *LcLdha* promoter after *LcHif-1α* overexpression in HEK293T after 12-h LPS stimulation **(A)**. Changes in transcription activity of *LcIl1β* promoter after LcHif-1α overexpression in HEK293T after 12-h LPS stimulation **(B)** (n = 3). LPS, lipopolysaccharide. “*” indicates P < 0.05,“**” indicates P < 0.01, and “***” indicates P < 0.001.

Dual-luciferase analysis showed that overexpression of *LcHif-1α* significantly increased the promoter activities of *LcLdha* and *LcIl1β* compared with the control group in HEK293T cells (**Figures 9A, B**). The results show that *Lc*Hif-1α activated the promoter activities of *LcLdha* and *LcIl1β*.

### 3.9 Comparative analysis of *Lc*HIF-1α, *Lc*COX2 protein levels, activities of key metabolic enzymes, and levels of metabolites and infectious factors in the head kidney of T and N after *A. hydrophila* infection

**Figure 10:**
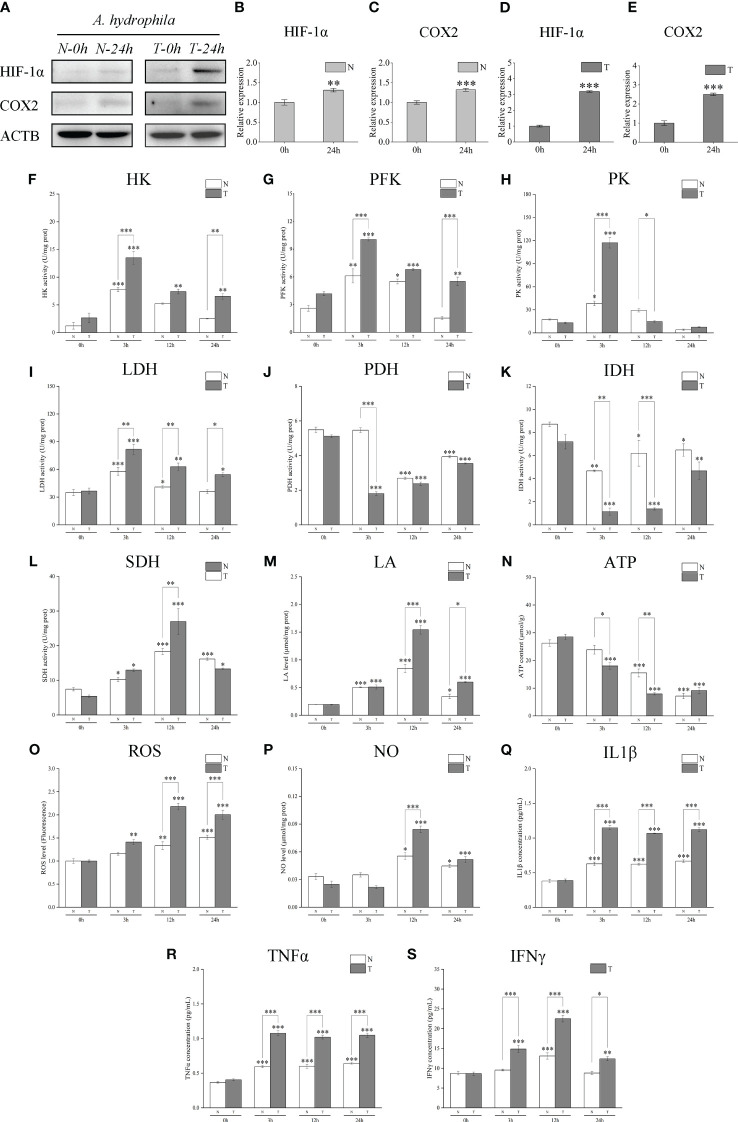
Changes of *Lc*HIF-1α and *Lc*COX2 protein, activities of the key metabolic enzymes, and levels of metabolites and inflammatory factors in the head kidney of hypoxia-tolerant population (T) and normal population (N) of *Larimichthys crocea* after *Aeromonas hydrophila* infection. Changes in *Lc*Hif-1α and *Lc*COX2 protein **(A–E)**; activities of HK **(F)**, PFK **(G)**, PK **(H)**, LDH **(I)**, PDH **(J)**, IDH **(K)**, and SDH **(L)**; and levels of LA **(M)**, ATP **(N)**, ROS **(O)**, NO **(P)**, IL1β **(Q)**, TNFα **(R)**, and IFNγ **(S)** in the head kidney of N and T after *A. hydrophila* infection for 24 h (n = 3). LPS, lipopolysaccharide; HK, hexokinase; PFK, phosphofructokinase; PK, pyruvate kinase; LDH, lactate dehydrogenase; PDH, pyruvate dehydrogenase; IDH, isocitrate dehydrogenase; SDH, succinate dehydrogenase; LA, lactic acid; ATP, adenosine triphosphate; ROS, reactive oxygen species; NO, nitric oxide. “*” indicates P < 0.05,“**” indicates P < 0.01, and “***” indicates P < 0.001.

Western blotting analysis showed that *Lc*HIF-1α and *Lc*COX2 proteins in the head kidney of N and T increased significantly after *A. hydrophila* infection and increased to a higher degree in T (**Figures 10A–E**). The determination of enzyme activity showed that the key glycolytic enzymes and metabolites also showed a similar dynamic trend: the activities of HK, PFK, PK, and LDH increased (**Figures 10F–I**); the yield of LA increased (**Figure 10M**); the activities of PDH and IDH decreased while the activities of SDH increased, with the T having a more significant trend than N (**Figures 10J–L**); ATP production was lower in T (**Figure 10N**). The bactericidal substances ROS and NO (**Figures 10O, P**) and pro-inflammatory factors IL1β, TNFα, and IFNγ showed an upward trend in both groups, and the degree of increase in T was also higher (**Figures 10Q–S**). These results indicate that T may have more or stronger metabolic transformation and polarization than the head and kidney macrophages in group N, and it may be caused by having more *Lc*HIF-1α.

## 4 Discussion

Recently, the decisive effect of metabolic reprogramming on the functional activities of macrophages has become an increasingly important research topic. Macrophages are relatively static in a stable state and react quickly following immune stimulation. This process usually involves numerous changes in gene expression to induce new functional abilities. Resting and activated states depend on different energy pathways. Activated macrophages show the so-called Warburg effect (also known as the glycolytic switch) for energy consumption, production of metabolites, and inflammatory factors (31–33). In this study, the glucose uptake gene *Glut1* expression was significantly increased after LPS stimulation. The glycolytic pathway enzymes PK, PFK, HK, and LDHA, which catalyze LA, gene mRNA expression, and enzyme activity, were increased, and the production of the pro-inflammatory glycolysis product LA was increased. These results may indicate that LPS leads to an increase in the glycolytic pathway flux of *L. crocea* head kidney macrophages (34). However, the yield of ATP was significantly reduced, which may be due to the lower energy efficiency of the glycolytic pathway, although ATP has a higher production rate without the electron transport chain (35). In addition, the M1-type macrophage metabolic checkpoint with the function of inhibiting PDH activity and pyruvate dehydrogenase kinase 1 (*Pdk1*) mRNA expression showed upregulation (36, 37). PDH, which is responsible for the conversion of pyruvate to acetyl-CoA for irreversible entry into the TCA cycle, was downregulated, and the mRNA expression and activity of mitochondrial IDH, an irreversible enzyme in the TCA cycle, were significantly reduced. Citric acid is normally converted into isocitrate by IDH and then into α-ketoglutarate (38). Therefore, the decreased activity of IDH in the TCA cycle of activated macrophages led to the first interruption of the TCA cycle (15); this is also called the M1 macrophage marker phenomenon (15, 36). These results may show that the *L. crocea* head kidney macrophages undergo metabolic reprogramming and show the Warburg effect after LPS stimulation.

In addition to glucose metabolism, the availability and metabolism of some amino acids also play an important role in regulating innate immunity. The three most well-studied amino acids include glutamine, arginine, and tryptophan (39). Glutamine can be used in amino acid and nucleotide synthesis, NADPH and energy production, and many other biosynthetic pathways involved in cell proliferation and function (40, 41). In M1-type macrophages, glutamine can be directly converted into α-ketoglutaric acid, a TCA intermediate, which is used as the key fuel of the TCA cycle after metabolic transformation and remodeling and is used by M1-type macrophages at high speed (15). Glutamine catabolism has been shown to play an important role in immune function against sepsis (42). Macrophages need sufficient glutamine to produce interleukin in response to LPS stimulation (43). Inhibition of glutaminase activity or removal of glutamine in the culture medium leads to the reduction of NO produced by activated macrophages (44, 45). In this study, the accumulation of glutamine in the LPS group was significantly lower than that in the CK group, which showed a high utilization of glutamine. This is similar to the results of the study on the changes in glutamine metabolism in *Coilia ectenes* infected by the Anisakidae parasite (46). The remodeled TCA cycle can be supplemented by arginine salt in addition to glutamine decomposition (15). The differential metabolism of arginine is one of the most reliable distinguishing factors between M1 and M2 macrophages (47). Different activated macrophages can induce different enzymes responsible for arginine metabolism. In M1 macrophages, nitric oxide synthase (iNOS) is upregulated, which leads to the decomposition of arginine into citrulline and nitric oxide, which can inactivate the electron transport chain in macrophages (48). The latter plays a key role in killing pathogens in cells. Arginase-1 (Arg1) is induced in M2 macrophages, which leads to the production of ornithine and promotes wound healing (47). In this study, arginine synthesis and metabolism in the LPS group decreased significantly, and ornithine accumulation also decreased significantly. In addition, the moderate catabolism of tryptophan is a sign of M1 macrophages (49). In our results, the accumulation of tryptophan in the LPS group decreased and the pathway of tryptophan biosynthesis decreased. Similar changes in tryptophan metabolism were also found in Atlantic salmon infected by *Aeromonas salmonicida* (50). The results showed that the amino acid metabolism of *L. crocea* head kidney macrophages stimulated by LPS participated in M1 polarization, which was similar to those in mammals. The increase of unsaturated fatty acids in cells will stimulate the production of inflammatory factors (51). Inflammatory stimulation can lead to the increase of fatty acid synthesis in macrophages (52, 53). This is very important for the differentiation and inflammatory function of macrophages (54). M2 macrophages rely on fatty acid oxidation, but the mechanism was not clear (55, 56). Here, the accumulation of glycerol and α-linolenic acid in the LPS group was significantly higher than the CK group, showing lipid accumulation, and the metabolites of steroid synthesis pathway were significantly upregulated, which may be related to the realization of the pro-inflammatory function of macrophages.

The mRNA expression level of *Inos* and the production of NO, an important bactericidal substance, were found to be significantly increased, which may be due to the changed metabolic state after the activation of macrophages, which tend to use arginine as the substrate of iNOS (57). ROS also increased significantly. As the TCA cycle was truncated, ROS production may be different from aerobic respiration, possibly promoted by increased SDH activity, mitochondrial succinic acid oxidation, and mitochondrial membrane potential (58). It has been shown that in mammalian macrophages incubated with LPS treated with glycolytic pathway inhibitor 2-deoxyglucose (2DG), the induction of the important inflammatory factor IL1β was blocked (19). Our results showed that the secretion of IL1β increased after LPS stimulation, which may also be regulated by metabolic reprogramming. Secretion of inflammatory mediator TNFα, mRNA level, and secretion of IFNγ were increased (8). Further, in the M1-type macrophage markers, *Cox2* mRNA expression increased, and COX2 protein accumulation was enhanced (8, 59). Our data indicated the *L. crocea* head kidney macrophages rapidly switched from the immune resting state to a highly active state and that the secretion of pro-inflammatory substances increased.

To date, it is still unclear how the metabolic transformation stimulated by LPS is regulated and how the metabolic transformation affects the polarization of *L. crocea* macrophages. Here, we investigated the expression of HIF-1α in *L. crocea* head kidney macrophages after LPS stimulation, and the results showed that HIF1-α mRNA expression and protein accumulation were increased independent of hypoxia. Our subcellular localization results also showed that HIF-1α was accumulated and transferred to the nucleus in *L. crocea* macrophages after LPS stimulation, similar to previous reports that HIF-1α must be transferred to the nucleus to regulate target genes (60). Observably, HIF-1α responded positively to LPS and may play a regulatory role in target gene regulation. To further clarify the regulation role of HIF-1α in metabolic reprogramming and functional activity, *LcHif-1α* was both interfered with and overexpressed. After the interference of *LcHif-1α*, the mRNA expressions of *Pdk1*, *Glut1*, *Pk*, *Pfk*, *Hk*, and *Ldha* were downregulated; the enzyme activities of PK, PFK, HK, and LDHA were inhibited; the production of LA was reduced, showing downregulation of the glycolytic pathway (61). The aerobic respiration-related enzymes PDH and IDH mRNA expression and activity increased, and the mRNA expression and activity of the pro-inflammatory gene SDH were downregulated. The reverse effect was observed when *Hif-1α* was overexpressed (39). Our results showed that HIF-1α played an important role in the metabolic pathway being transformed to aerobic glycolysis in the *L. crocea* head kidney macrophages under LPS stimulation.

In addition to the effect of HIF-1α on LA, a pro-inflammatory metabolite, the production of ROS and NO was inhibited after *Hif-1α* interference. This may be because the production of ROS in LPS-induced macrophages was affected by SDH activity (58). The regulation of NO may be related to the fact that *Inos* is the target gene of HIF-1α, and the increased utilization of arginine, the substrate of iNOS, after metabolic transformation (62). This also corresponds to the decreased SDH mRNA level and activity and decreased *Inos* mRNA level. In addition, mRNA levels and secretion of IL1β and IFNγ, reportedly regulated by HIF-1α, were decreased, as well as TNFα (19, 63, 64). The level of M1-type macrophage markers COX2 was also downregulated, showing a weaker degree of polarization (8, 65–67). In contrast, *LcHif-1α* overexpression produced the opposite results. Furthermore, dual-luciferase experiments showed that the overexpression of *LcHif-1α* could significantly enhance the promoter activities of the glycolytic enzyme *Ldha* and inflammatory factor *Il1β*. HIF-1α was involved in the drive of metabolic reprogramming to glycolysis, which may result in the production of bactericidal substances against pathogens, promoting the polarization of macrophages. Studies have shown that the specific deletion of HIF-1α could reduce the production of ATP, thereby adversely affecting the migration, invasion, and phagocytosis of macrophages (68–70). Our results are consistent with previous research: *LcHif-α* interference resulted in a decrease in ATP, and the migration, invasion, and phagocytosis of macrophages were decreased, which showed the opposite trend when *LcHif-1α* was overexpressed. In addition, through the observation of ultrastructure in this study, it was found that after si*LcHif-1α* and siNC, there were still many micro-folds and protrusions on the surface of *L. crocea* head kidney macrophages, and the nucleus was deeply colored, which was similar to normal macrophages (8). After stimulation by siNC and LPS, the nuclear membrane of macrophages was smoother and showed a healthier state, fewer mitochondria, and more lysosomes, phagocytes, and endoplasmic reticulum in cytoplasm, which are in line with the characteristics of functional macrophages (71, 72). There were fewer engulfed beads in the phagocyte of macrophages after *LcHif-1α* interference. It can be seen that *LcHif-1α* interference reduced the phagocytic function of macrophages. In addition, cell damage can be characterized by severe nuclear membrane depression (73), mitochondrial elongation, breakage, and degradation (74). The damaged organelles may be wrapped by intracellular membranes to form autophagy and then fuse with primary lysosomes to form autolysosomes (75). After *LcHif-1α* interfered, the nuclear membrane of *L. crocea* head kidney macrophages was seriously depressed; the mitochondria were deformed, elongated, and folded; some mitochondrial ridges disappeared and autolysosomes were produced; these may indicate that macrophage was damaged after *LcHif-1α* interference and that *LcHif-1α* may play a protective role in cell damage (29). These results suggested that HIF-1α drives metabolic reprogramming and enhances the functional activity of *L. crocea* head kidney macrophages (76).

**Figure 11:**
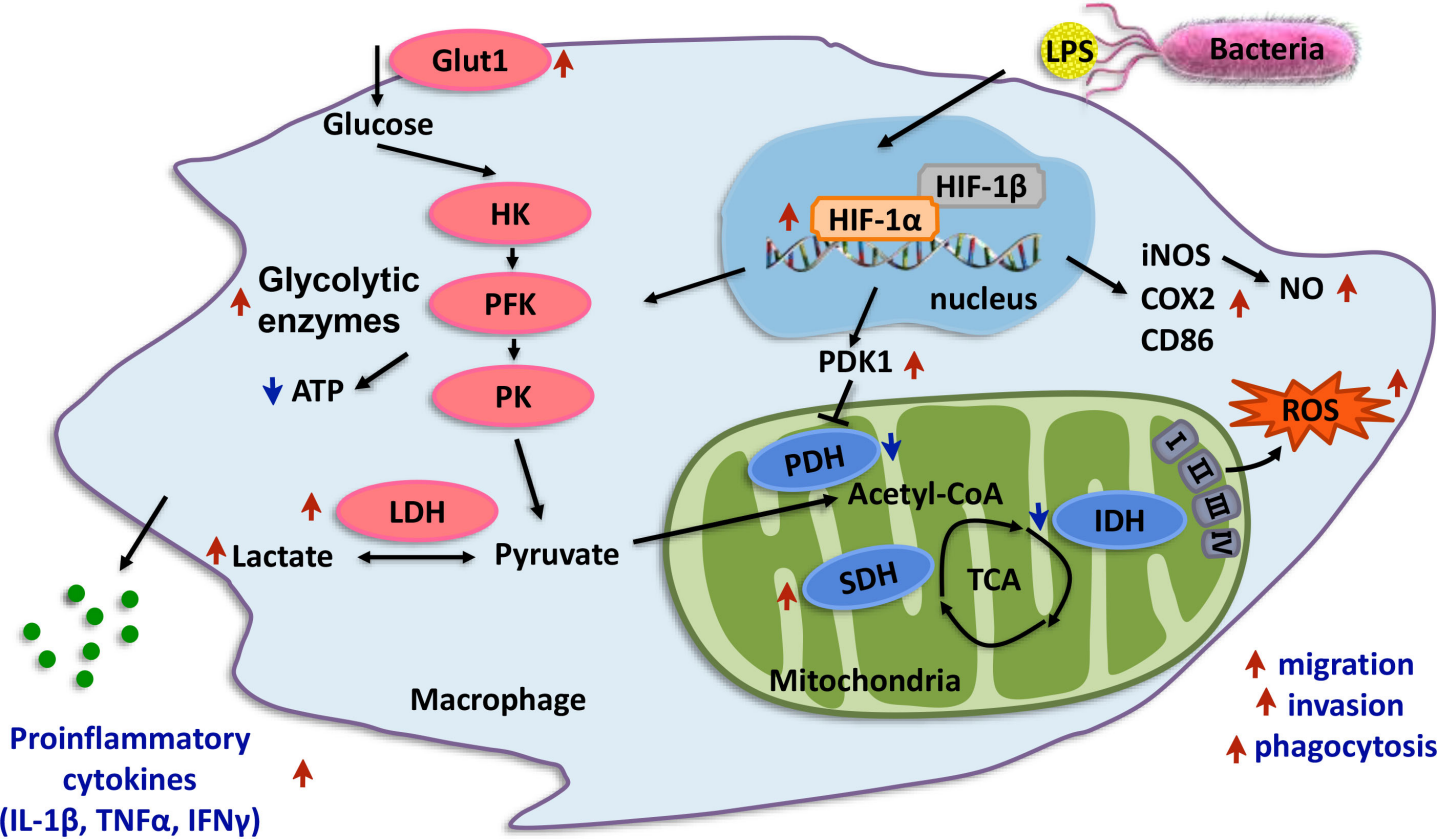
Proposed working model of HIF-1α promotes macrophage functional activities in protecting hypoxia-tolerant *Larimichthys crocea* against *Aeromonas hydrophila* infection. *Lc*HIF-1α induced by LPS; LPS leading to the nuclear translocation of *Lc*HIF-1α, which promotes levels of glycolytic pathway enzymes and glycolytic metabolites and inhibits the aerobic respiratory enzymes, making macrophages show Warburg effect. *Lc*HIF-1α enhances levels of bactericidal products, inflammatory factors, and M1-type maker genes and plays a role in migration, invasion, and phagocytosis of macrophages to achieve its M1 polarization state and sterilization ability. *Lc*HIF-1α controls the transcription of *LcLdha* and *LcIl1β* by binding to their promoter. *Lc*HIF-1α may be involved in the metabolic transformation of macrophages in the head kidney of the T of *L. crocea* and promote its functional activity and defense against *A. hydrophila* infection. The red arrows indicate the enhancing effects, and the green arrows show the suppressing effects.

The effect of HIF-1α on immunity is not limited *in vitro*. Previous studies have reported that HIF-1α plays a key role in bacterial resistance in mice and zebrafish and improves the survival rate (77, 78). In our study, at 24 h after *A. hydrophila* infection, the N and T mortality rates were significantly different, and the HIF-1α protein in both N and T was increased compared with 0 h. However, the increase in T at 24 h from T at 0 h in the head kidney was significantly greater than that in N, which was consistent with the increase in *Hif-1α* mRNA expression in our previous study (23). The increased accumulation rate of COX2, a type of M1 macrophage marker, also showed the same trend (79). In addition, the activities of PK, PFK, HK, and LDHA in T were increased higher than in N, and the yield of ATP was lower. The activities of the aerobic respiratory enzymes PDH and IDH decreased. The SDH activity; levels of the bactericidal substances LA, ROS, and NO; and secretion of the inflammatory factors IL1β, TNFα, and IFNγ were significantly increased (80, 81). These findings demonstrate that the HIF-1α may be involved in the metabolic transformation of immune cells in the head kidney of the T of *L. crocea*, promote its functional activity and defense against *A. hydrophila* infection, and may contribute to the improvement of the survival rate of hypoxia-tolerant *L. crocea* (82, 83) (Figure 11).

## Data availability statement

The original contributions presented in the study are included in the article/**Supplementary Material**. Further inquiries can be directed to the corresponding authors.

## Ethics statement

The animal study was approved by Animal Care and Use Committee of Ningbo University. The study was conducted in accordance with the local legislation and institutional requirements.

## Author contributions

YZ: Conceptualization, Data curation, Investigation, Methodology, Software, Writing – original draft, Writing – review & editing. XLW: Conceptualization, Data curation, Supervision, Writing – review & editing. JD: Data curation, Software, Writing – review & editing. ZG: Data curation, Investigation, Software, Writing – review & editing. XL: Methodology, Supervision, Writing – original draft. RM: Investigation, Software, Validation, Writing – review & editing. XFW: Funding acquisition, Project administration, Resources, Supervision, Visualization, Writing – review & editing. WS: Funding acquisition, Project administration, Resources, Supervision, Visualization, Writing – original draft, Writing – review & editing. JZ: Project administration, Resources, Supervision, Validation, Visualization, Writing – original draft, Writing – review & editing.
