# Supplementary material for: Hypoxia-inducible factor-1α promotes macrophage functional activities in protecting hypoxia-tolerant large yellow croaker (Larimichthys crocea) against Aeromonas hydrophila infection
Source: Front Immunol. 2024 Aug 2;15:1410082. doi: 10.3389/fimmu.2024.1410082 (PMC11327042; doi:10.3389/fimmu.2024.1410082)
Supplement: Supplementary file 1 [file DataSheet_1.docx]

Supplementary Material

# Supplementary Figures and Tables

For more information on Supplementary Material and for details on the different file types accepted, please see [here](https://www.frontiersin.org/guidelines/author-guidelines#supplementary-material).

## Supplementary Figures


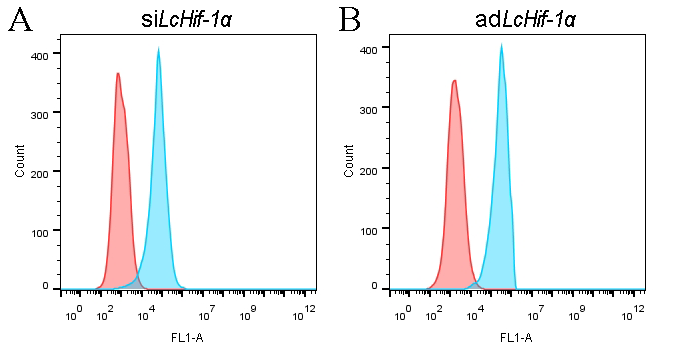


**Supplementary Fig. S1.** The interference and overexpression efficiency of LcHif-1α were shown in the figure.


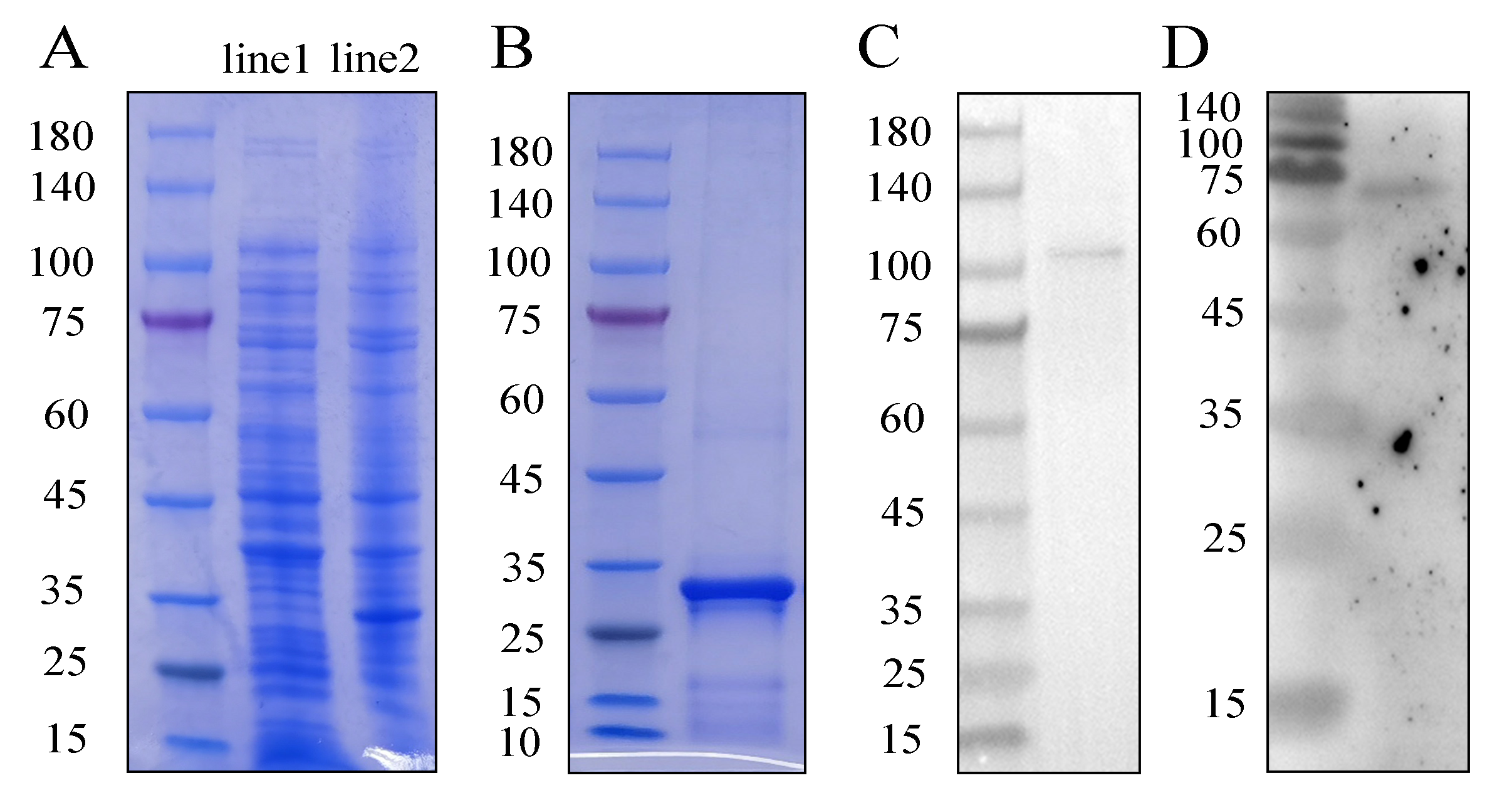


**Supplementary Fig. S2.** (A) Expression and purification of recombinant protein and specific analysis using mouse anti-*Lc*HIF-1α antibodies. Line 1 shows the total protein of DE3-*Lc*HIF-1α without IPTG induction. Line 2 shows total DE3-*Lc*HIF-1α induced with IPTG. (B) purified recombination protein. (C) The specificity of *Lc*HIF-1α. Western blot (WB) analysis shows only a single protein band was detected. (D) The specificity of COX2 validated by WB.





**Supplementary Fig. S3.** Expression patterns of metabolic and immune related genes in *L. crocea* head kidney macrophages after *LcHif-1α* overexpression following 12 h LPS stimulation. mRNA expression of *LcGlut1* (A), *LcHk1* (B), *LcPfk* (C), *LcPkm* (D), *LcLdha* (E), *LcPdk1*(F), *LcPdh* (G), *LcIdh* (H), *LcSdh* (I), *LcCox2* (J), *LcIl1β* (K), *LcTnfα* (L), *LcIfnγ* (M) and *LcInos* (N) in macrophages after *LcHif-1α* overexpression following 12 h LPS stimulation.





**Supplementary Fig. S4.** Detection of the activity of the key metabolic enzymes, and levels of metabolites and inflammatory factors in *L. crocea* head kidney macrophages after *LcHif-1α* overexpression following 12 h LPS stimulation. Changes in HK (A), PFK (B), PK (C), LDH (D), PDH (E), IDH (F), and SDH (G) activity, and LA (H), ATP (I), ROS (J), NO (K), IL1β (L), TNFα (M) and IFNγ (N) levels in macrophages after *LcHif-1α* overexpression following 12 h LPS stimulation.

## Supplementary Table

Supplementary Table S1. Primers used in this study.

| Primer name | Primer sequence (5′-3′) | | Used for |
| --- | --- | --- | --- |
| *LcHif-1α*-qF | CTTCCTCAGCCGCCATACA | real-time PCR | |
| *LcHif-1α*-qR | CACAGAACGATTCAACAGGTCA | real-time PCR | |
| *LcGlut1*-qF | GCCTTGGCTCTGCTGGAACAAT | real-time PCR | |
| *LcGlut1*-qR | GCCTGGGTCCCTGAGAGAACAA | real-time PCR | |
| *LcHk1*-qF | GGAGGATTACCCCTGAACTGC | real-time PCR | |
| *LcHk1*-qR | CCTTGGACAACCCCTCCTTACT | real-time PCR | |
| *LcPfk*-qF | GATGAACGCTGCTGTCCGA | real-time PCR | |
| *LcPfk*-qR | GTGCCAGTTTGATGTTGTCTCC | real-time PCR | |
| *LcPkm*-qF | GGCAGAAGCGGCCATTTT | real-time PCR | |
| *LcPkm*-qR | CAGCACAGCATTTGAAGGAGG | real-time PCR | |
| *LcLdha*-qF | AAGTTCATCATCCCAAACATCG | real-time PCR | |
| *LcLdha*-qR | CAGGTTGGTGCCAGAGCC | real-time PCR | |
| *LcPdk1*-qF | GCTCAGGGAGTCGTCGAGTACA | real-time PCR | |
| *LcPdk1*-qR | CCGAAGAGGAGAGTGTGCTGGT | real-time PCR | |
| *LcPdh*-qF | CGAGACCAGCGTGATGAAGACC | real-time PCR | |
| *LcPdh*-qR | GAGCGTCCAGGTAGTTGAAGGC | real-time PCR | |
| *LcIdh*-qF | GGTGGTGGAGATGGACGGAGAT | real-time PCR | |
| *LcIdh*-qR | TGGTCATCTGTCTGGTCACGGT | real-time PCR | |
| *LcSdh*-qF | TGCCAGTGTCCACGGTGCTAA | real-time PCR | |
| *LcSdh*-qR | TGTGCTCCTCGGTGATGGTGAG | real-time PCR | |
| *LcCox2*-qF | GTCCCCGGTCTGATGATGTAT | real-time PCR | |
| *LcCox2*-qR | GGTCTGGAAGAGCCTTTCGT | real-time PCR | |
| *LcIl1β*-qF | AGAGGAGGAAACTGTGAACGC | real-time PCR | |
| *LcIl1β*-qR | TGTTGGTGATGGACAGGACG | real-time PCR | |
| *LcTnfα*-qF | ACACCTCTCAGCCACAGGAT | real-time PCR | |
| *LcTnfα*-qR | CCGTGTCCCACTCCATAGTT | real-time PCR | |
| *LcIfnγ*-qF | GGACTGCATCGCCTCAAACACA | real-time PCR | |
| *LcIfnγ*-qR | TGCTTCTCCTTCCTGGTGCTGT | real-time PCR | |
| *LcInos*-qF | ACTCGTCAATCCGACCCTG | real-time PCR | |
| *LcInos*-qR | CGCACACTTTGGTCATCAG | real-time PCR | |
| *Lcβ-actin-*qF | GTTATGCCCTGCCCCATG | real-time PCR | |
| *Lcβ-actin-*qR | TGTCACGCACGATTTCCCT | real-time PCR | |
| *siLcHif-1α*-F | CGCACCGUCAAUGUCAAAUTT | RNAi | |
| *siLcHif-1α*-R | AUUUGACAUUGACGGUGCGTT | RNAi | |
| siNC-F | UUCUCCGAACGUGUCAGGUTT | RNAi | |
| siNC-R | ACGUGACACGUUCGGAGAATT | RNAi | |
| *Lchif-1α*-pcDNA3.1-F | CGCGGATCCATGGACACAGGAAT  TGTACCAGAAAAGAAA | Overexpression | |
| *Lchif-1α*-pcDNA3.1-R | CCGCTCGAGAATGACGTGGTCCAGAGCGC | Overexpression | |
| *LcHif-1α*-F | cagcaaatgggtcgcggatccCTCAATGGCTCCTACCTAAAGGC | Recombinant expression | |
| *LcHif-1α*-R | gtggtggtggtggtgctcgagGTAGATGACAGTGGCTTGGGTTT | Recombinant expression | |
| *LcIl1β*-pGL3-F | AGGTACCGAGCTCTTACGCGTCACTACTTTTCTGCTTACCTATTAAATGTT | Luciferase reporter | |
| *LcIl1β*-pGL3-R | ACTTAGATCGCAGATCTCGAGGTGAAGTGCGTTTACCTGTTAAGG | Luciferase reporter | |
| *LcLdha*-pGL3-F | ATTTCTCTATCGATAGGTACCCTGGATAATC  TGAGCACGAAATTT | Luciferase reporter | |
| *LcLdha*-pGL3-R | CAGTACCGGAATGCCAAGCTTGGATGCGGC  GATGAGACA | Luciferase reporter | |
